# Physiotherapy Management in the Case of Primary Peritoneal Serous Carcinoma With Lower Segment Cesarean Section: A Case Report

**DOI:** 10.7759/cureus.53903

**Published:** 2024-02-09

**Authors:** Sphurti S Bhoite, Neha V Chitale, Tushar J Palekar

**Affiliations:** 1 Department of Physical Medicine and Rehabilitation, Dr. D. Y. Patil College of Physiotherapy, Dr. D. Y. Patil Vidyapeeth, Pune, IND; 2 Department of Musculoskeletal Physiotherapy, Dr. D. Y. Patil College of Physiotherapy, Dr. D. Y. Patil Vidyapeeth, Pune, IND

**Keywords:** oncology case report, physical therapy rehabilitation, primary peritoneal serous carcinoma, molluscum contagiosum virus, lower segment caesarean section

## Abstract

This case report presents the medical path of a 24-year-old female patient, who had undergone lower (uterine) segment cesarean section (LSCS) while facing complications of having several diagnoses at the same time, including primary peritoneal serous cancer, sexually transmitted disease (STD), and IgM-positive dengue. The prevention and treatment of STDs require an integrated approach due to the persistent problems they provide in the global healthcare system. In India, there is a high birth rate, which makes LSCS a common treatment. The combination of dengue fever, STDs, cancer care, and such issues related to women’s health emphasizes the necessity of specialized interventions to reduce the risk of problems both during and after pregnancy. A sophisticated, multidisciplinary approach to postoperative care is required due to the confluence of these disorders, with physiotherapy and rehabilitation serving as a crucial treatment approach. The patient received breathing exercises along with core strengthening exercises. For relaxation, Benson's relaxation technique was used. Significant improvement was seen in the patient's muscle strength and quality of life post rehabilitation.

## Introduction

In India, despite having the largest population, people have always avoided talking about sexual intimacy or any related topic. It has been associated with a sense of unease and internalized shame for ages. There are several causes for this, the most significant of which are sociocultural causes. The traditional outlook of Indian households prevents healthy conversations about adolescence and sexuality. When it comes to sex education, the Indian curriculum is found to be seriously insufficient. The most typical situation involves having several partners who are also obligated to sexually transmitted diseases (STDs), which are infections that are spread from person to person by intimate sexual contact, such as oral, vaginal, or anal sex [1]. Lack of awareness of contraceptive devices, such as condoms, is also a cause of STDs, also known as sexually transmitted infections. Molluscum contagiosum, another name for water warts, is a benign cutaneous condition. Molluscum contagiosum is the source of skin lesions known as "Mollusca." The typical lesion is dome-shaped, round, and pinkish-purple in color. This practice looks at the causes and symptoms of molluscum contagiosum and highlights the need for an interprofessional team in treating the illness [2].

An incision through the abdominal wall and uterus, as opposed to the pelvis and vagina, is used to deliver the baby during a lower segment cesarean section (LSCS). It can be administered as general, spinal, or epidural anesthesia, respectively. A cesarean section can be performed using a transverse (side-to-side) or vertical (up-and-down) incision. The kind of incision done, nevertheless, depends on the mother's and the fetus's conditions. Complications of the LSCS may be due to above-average blood loss, blood clots, infection in the lining of the uterus, and postpartum hemorrhage [3].

With a prevalence of 7% in women initially diagnosed with ovarian cancer, primary peritoneal serous carcinoma (PPSC) is an uncommon malignancy characterized by abdominopelvic carcinomatosis with no or minimal surface involvement of the ovaries [4]. Clinically and histopathologically, it is impossible to distinguish between primary serous ovarian cancer and PPSC, a rare primary tumor diffusively involving the peritoneum. Primary peritoneal cancer was first described by Swerdlow in 1959. It is unclear what exactly causes PPSC. The peritoneum and the ovary share a similar embryonic genesis, as both epithelial layers develop from coelomic epithelium early in life. The precise etiology of peritoneal cancer is unresolved, yet out-of-control spread in peritoneal cells is the first step in the process. This may be associated with another type of cancer called primary peritoneal cancer, or with a prior malignancy that has now spread to the peritoneum [5].

In postpartum females, muscle strength along with pelvic floor strength remains ignored, which in the future affects the quality of life of the individual. Physiotherapy rehabilitation protocol helps in maintaining as well as increasing muscle strength. Benson's relaxation technique is the method that helps in deep relaxation and leads to early recovery of the muscles. In this case, we have given strength training and breathing exercises along with relaxation techniques, and post-rehabilitation quality of life and muscle strength were assessed.

## Case presentation

A 24-year-old woman was admitted to the Department of Gynecology of Dr. D. Y. Patil Hospital by reference from a local hospital with complaints of pain and an amniotic fluid index of 6 cm (oligohydramnios). The patient was a known case of PPSC, in addition to being dengue-positive and having molluscum contagiosum. On assessment, the resisted isometric grading of the muscles was weak and painful (Table 1).

**Table 1:** Resisted isometric grading

Resisted isometric grading	Pre	Post
Multifidus	Weak and painful	Weak and painless
Erector spinae	Weak and painful	Weak and painless
Rectus abdominis	Weak and painful	Strong and painful
Transverse abdominis	Weak and painful	Strong and painful
Serratus anterior	Weak	Strong

Rehabilitation protocol

One to Two Weeks

Rest: Rest whenever possible and keep all belongings of the mother and the baby nearby. Limit any heavy lifting, which can further cause pain and discomfort to the mother. Limit excessive walking or other activities of daily living.

Adequate water intake: Consuming adequate fluid will help prevent dehydration and constipation. In the phase of breastfeeding, there is a higher demand for thirst, so water intake should be increased for hydration.

Nutritious dietary intake: The most common postpartum discomfort felt is constipation. Due to the cesarean section, pain medication, prenatal vitamins, and essential medicines can give rise to abdominal discomfort. Along with water intake, sources of fiber and protein should be inculcated. Choosing iron-rich foods can help maintain hemoglobin levels and reduce the risk of iron deficiency anemia.

Initiation of Relaxation Techniques

Benson’s relaxation therapy: Many new mothers experience discomfort, mood swings, postpartum depression, and stress after a cesarean procedure. Because non-pharmacological techniques are easy to apply, affordable, and free of side effects, they can be used to ease these discomforts. Benson's relaxation therapy (BRT), which is breathing in and out while sitting with closed eyes and uttering the word "one" to oneself as you exhale, is a recommended breathing exercise for new mothers.

Segmental breathing: Lateral basal expansion, or unilateral or bilateral lateral costal expansion, is a possible technique. Deep breathing and paying attention to the movement of the lower rib cage can help to promote the diaphragmatic excursion. This aids in upper trunk relaxation.

Thoracic expansion: Larger-than-normal breaths are taken during aggressive inspirations, which are followed by relaxed expiration. During expiration, this causes an increase in airflow through the collateral ventilation channels and peripheral airways. Hands should be expanded and elevated during inhaling and lowered during exhalation.

Splinting: Splinting can be used to immobilize during the early phases of wound healing and, in conjunction with passive or active stretching, can be used to alter the alignment of collagen. Over time, elongation and stretching of tissues can occur due to changes in the viscoelastic characteristics of tissues caused by static and dynamic splinting. Make use of a tiny pillow, towel, or receiving blanket that is close by. The material should be folded such that it reaches past the length of the incision and is roughly two-hand widths high. Press the towel (or pillow or blanket) over the wound with flat palms, pressing firmly and evenly all over the incision. Breathe in while maintaining the splint in place, and then out slightly while moving or doing your activity.

Breastfeeding ergonomics: It can be exhausting to sit for long lengths of time when nursing, so having a chair with the right back support is crucial. When leaning back in the chair, make sure the lower back is properly supported. If there are gaps between the chair and your back, place a pillow behind you or a rolled towel there. Finding a chair that enables you to stay with both feet flat on the floor while bending your hips and knees to about an angle of 90 degrees is also great. To ease the tension on the baby, try using cushions to support them rather than shoulders, arms, or wrists. Place the cushions on the baby's level so that they are level with the breast.

Two to Four Weeks

Light aerobic activity: Walking, swimming, cycling, and aerobic training are light aerobic workouts. Gradually boost the intensity of your workouts as your strength and endurance improve.

Static core activation: (1) Static back: The lower back joints are in their most open posture when the hips and knees are raised to a 90-degree angle. Because there is less weight and strain on the lower back in this open position, all of the structures can relax. With a static back, you put your shoulders in the same plane as your hips and use gravity and body weight to gradually and passively release your lower back muscles. Additionally, static back promotes mid-spine extension and activates the hip flexors. Your upper back can start to expand up against the floor's smooth surface in this position. It is important to breathe deeply through the belly while in this position. (2) Static hamstrings: Start this exercise by bending the knee while lying supine. Tighten the hamstrings (the back of your thigh) by pressing the heel into the bed. Hold for five seconds then relax. (3) Static quadriceps: On the unaffected leg, begin to strengthen the quadriceps, push the knee into the bed, and pull the toes up toward the shin. The heel should rise off the bed a little as a result of this. Hold for five seconds, then relax.

Four to Six Weeks

Strength training - transverse abdominis activation: The abdominal wall's deepest layer is called the transverse abdominis. Given that these muscles cooperate with the pelvic floor, a pelvic floor muscular contraction, or Kegel, is an excellent approach to activate these muscles following a cesarean delivery.

Ball squeeze: Contracting the inner thigh muscle engages the transverse abdominis muscles and the pelvic floor muscles. This ball squeeze exercise can be conducted either seated with feet on the floor or lying down on the back as a fast and efficient means to begin strengthening your core.

Pelvic bridging: Exercises with pelvic control require the use of the core muscles, like the multifidus and transverse belly muscles, in addition to the hip extensors and abductors. Contract the inner thigh muscle to activate the transverse abdominis and pelvic floor. For an easy and efficient way to start engaging your core, you can perform this ball squeeze exercise either lying down on your back or while seated with your feet on the floor. Lying flat on your back with your knees bent and your feet resting on the floor, take a deep breath. Press through your heels to lift your hips off the ground and exhale. In the erect position, squeeze your buttocks and gently draw in your lower abdomen and pelvic floor. Repeat after inhaling once more.

Kegel exercise: Your pelvic floor can be effectively engaged and strengthened with Kegel exercises. They reduce stress-related postpartum incontinence, as evidenced by studies. These activities will be beneficial when the urine catheter is withdrawn, which is a post-C-section necessity. Place your feet on the floor and take a seat on a chair's edge. Tighten your pelvic floor muscles. You should feel as though you are attempting to stop the pee flow. Envision shutting off the urethra, anus, and vagina completely. Consider lifting them out of the chair as well. Keep this contraction going for a bit. Increase the time by five seconds at first. Breathe in deeply, then relax the contraction by exhaling fully. Perform some exercises with Kegel in different derived positions. Perform 10 to 12 times with a one-minute rest between contractions. Facilitation using facilitator techniques for weak muscles (muscle tapping, muscle activation, vibration, isometric, eccentric, and concentric contraction).

Stretching calf muscles: The therapist stretched the patient's calf muscles while the patient lay supine. The therapist cupped the patient's heel and did a dorsiflexion of the foot while supporting the other hand above the knee to prevent flexion.

Scar mobilization: As it helps to prevent adhesions and promote collagen production, massage is advantageous to collagen synthesis during the proliferative period. Collagen realignment is aided by the mechanical stress that is also imparted to the intermolecular connections (Table 2).

**Table 2:** Rehabilitation protocol

Weeks	Physiotherapy management
1-2 weeks	Rest, adequate water intake, nutritious dietary intake, initiation of relaxation technique, log rolling in and out of bed, splinting (supporting with incision with hands), ergonomics for breastfeeding
2-4 weeks	Light aerobic activity (walking at own pace), improvements of activities of daily living, static core activation
4-6 weeks	Progress aerobic exercise, strength training (including pelvic floor muscles), stretching exercises for the shortened muscles, facilitation using facilitator techniques for weak muscles (vibration, muscle activation muscle tapping), isometric, eccentric, and concentric contraction, scar mobilization
Post 6 weeks	Increase tolerance to aerobic exercise, strength training of core muscles

Follow up

Pelvic floor muscle assessment (PFM) was assessed using vaginal cones before physiotherapy rehabilitation, and the results showed a score of 3 (moderate). Post rehabilitation, the PFM score was 4 (good). There is increased tension and a strong contraction that can raise the posterior vaginal wall against opposition.

The Numerical Pain Rating Scale (NPRS) was assessed prior, which revealed an NPRS score of 7, and post rehabilitation, the NPRS score was 1.

Maternal postpartum quality of life was assessed before physiotherapy rehabilitation. The score was 50, and the rehabilitation score was 65. There was a significant increase in the postpartum quality of life (Table 3). Therefore, improvement was seen in the condition of the patient.

**Table 3:** Follow-up post treatment

Outcome measures	Pre-rehabilitation	Post-rehabilitation
Numerical Pain Rating Scale	7	1
Pelvic floor muscle assessment	Moderate = 3	Good = 4
Maternal postpartum quality of life	50 score	65 score

## Discussion

This case report is of a 24-year-old female, who was a known case of PPSC, in addition to being dengue IgM-positive and having molluscum contagiosum. Pain relief and strengthening of the weak muscles were the two main objectives of rehabilitation. She had given birth a few days prior; therefore, her weak core muscles were the source of her agony. Exercises to maintain strength in the pelvic floor muscles and core muscles should be initiated for long-term benefits.

In 2016, Yun et al. [6] documented a case that was investigated by computed tomography (CT) to study PPSC, an incredibly rare malignancy that showed numerous ascites in the abdominal cavity and omental infiltration [6]. The case was operated and medically managed. The patient was initially in agony during physiotherapy treatment, but the pain progressively decreased and, after a few sessions, the patient's core strength and ability to do daily activities significantly improved.

A randomized controlled trial (RCT) involving 54 women who had LSCS was carried out in 2022 by Weerasinghe et al. [7] to investigate the efficacy of physiotherapy post-LSCS and preoperative education. They concluded that post-physiotherapy rehabilitation was beneficial when paired with earlier physical rehabilitation. R Velingkar et al. [8] conducted a study in 2022 on the effects of transcutaneous electrical nerve stimulation (TENS) on pain intensity and functional activities following LSCS. The study used the Patient-Specific Functional Scale (PSFS) and the NPRS to measure postoperative incisional pain. The researchers found that administering TENS after LSCS improved pain intensity and functional activities as measured by the NPRS and PSFS, respectively.

Based on the evidence reviewed above, it appears that the majority of women who had LSCS benefited from physiotherapy rehabilitation. The regimen for rehabilitation was divided into four phases. In the initial post-LSCS period, the patient was advised to rest and avoid activities that might exacerbate her pain or discomfort. She was encouraged to rest, adhere to a postpartum diet, and practice good ergonomics during the first two weeks after giving birth. During the second and fourth weeks of recovery, light aerobics and core activation were administered. In addition, strength training and aerobic progression were carried out over the next four to six weeks. After six weeks, there was a rise in the intensity of the exercises and a greater advancement in strength training.

A traditional rehabilitation regimen was established for this case with the goals of lowering discomfort and strengthening the pelvic floor and core muscles. Strength training helps the patient do functional activities more easily and lowers weakness, which is crucial for preventing subsequent difficulties.

## Conclusions

This case study provides a comprehensive exploration of the intricate interplay between the various diagnoses mentioned, emphasizing the need for personalized and integrated healthcare strategies. The success observed in the physiotherapy management of this complex case advocates for the consideration of such interventions in the postoperative care of women undergoing LSCS. The findings encourage further research to validate and expand on integrated approaches, solidifying the role of physiotherapy as a valuable and effective treatment modality in the postoperative care of women facing complex medical scenarios.
